# Desensitization for Vitamin B12 Hypersensitivity and How to Do It

**DOI:** 10.3390/biomedicines13040801

**Published:** 2025-03-26

**Authors:** Kinga Lis

**Affiliations:** Department of Allergology, Clinical Immunology and Internal Medicine, Ludwik Rydygier Collegium Medicum in Bydgoszcz, Nicolaus Copernicus University in Torun, ul. Ujejskiego 75, 85-168 Bydgoszcz, Poland; kinga.lis@cm.umk.pl

**Keywords:** vitamin B12, cobalamin, hypersensitivity, desensitization

## Abstract

Vitamin B12 is the common name for a group of cobalamins, which are cobalt corrines. Cobalamins are water-soluble B vitamins. Vitamin B12, as a coenzyme of various enzymes, is an essential component of many key metabolic processes in the body. Vitamin B12 deficiency causes dysfunction of various organs and systems in the body, including the central nervous system. Humans, like other animals, are unable to synthesize cobalamin. This vitamin must be supplied with a balanced diet. The only valuable dietary sources of cobalamin are foods of animal origin, especially offal (e.g., liver). Vegan and vegetarian diets are deficient in vitamin B12. People who follow this nutritional model require systematic cobalamin supplementation, usually in oral form. Other causes of cobalamin deficiency are various pathogenetic processes, in the course of which any of the stages of the complicated process of absorption of this vitamin from the gastrointestinal tract are impaired. Disorders of intestinal absorption of vitamin B12 require systematic supplementation of cobalamin parenterally (usually by intramuscular or subcutaneous injections) for the rest of life. Supplementary therapy with vitamin B12 may cause various adverse reactions, among which hypersensitivity reactions of various spectrums and intensity of symptoms are possible. According to available data, hypersensitivity to cobalamin is more likely after intramuscular or subcutaneous administration than in oral form. It also seems that long-term administration of cobalamin predisposes to allergy to vitamin B12, regardless of its chemical form. Although hypersensitivity to cobalamin is rather rare, it can also be of great clinical importance. This is due to the fact that vitamin B12 deficiency affects a significant part of the population, especially the elderly and those with chronic diseases that impair its absorption. In addition, supplementary therapy with cobalamin is long-term (usually lifelong) and there is no alternative form of treatment. For these reasons, solutions are sought that will allow for the safe continuation of treatment supplementing cobalamin deficiency. Various cyanocobalamin desensitization protocols are proposed, differing in duration, the dynamics of gradual dose increase, or the method of injection (intramuscular or subcutaneous). An analysis of available data in this field suggests that desensitization with cyanocobalamin seems to be an effective way to obtain tolerance to vitamin B12, allowing for long-term supplementation of this vitamin regardless of the chemical form, dose size, frequency, or route of administration.

## 1. Introduction

Vitamin B12 is a substance that must be supplied to the body with food because it cannot be synthesized by humans. As a coenzyme of key enzymes in the body, it is essential for the proper functioning of many different organs and tissues. Vitamin B12 deficiency results in the dysfunction of numerous systems, including the central nervous system [1]. Only bacteria and archaebacteria are capable of synthesizing vitamin B12 [2]. Although the human colonic microflora is able to produce cobalamin, this synthesis takes place outside the place where vitamin B12 is absorbed from the digestive tract. It is therefore inaccessible to the body. Excess vitamin B12 absorbed from food accumulates in animal tissues. Plants do not produce cobalamin. Therefore, the only source of this vitamin in the diet is animal products [3]. This means that people on diets that exclude meat and offal require constant supplementation with vitamin B12, while the lack of such intervention will lead to its deficiency and the resulting complications [4]. The second group of people at risk of vitamin B12 deficiency are people whose cobalamin absorption processes from the gastrointestinal tract are impaired. Since vitamin B12 absorption is a very complex and multi-stage process, the probability of its dysfunction is high, it can have various pathogenetic backgrounds and be the result of many diseases with diverse causes [5,6]. People with impaired vitamin B12 absorption require a supply of this vitamin, usually parenterally (intramuscular or subcutaneous injections) for the rest of their lives [5,6].

Any medical intervention requiring drug administration can lead to various adverse effects, including hypersensitivity reactions [7]. Hypersensitivity to vitamin B12 is considered a relatively rare phenomenon. Despite this, it can be a significant problem due to the fact that cobalamin therapy is the only effective therapeutic option in some clinical conditions [8]. For this reason, attempts are being made at cobalamin desensitization and the results of these interventions seem to be effective [9,10,11,12,13,14,15,16].

This review focuses on the analysis of the problem of vitamin B12 hypersensitivity and presents different protocols for cyanocobalamin desensitization based on available literature data.

## 2. Vitamin B12

**Figure 1:**
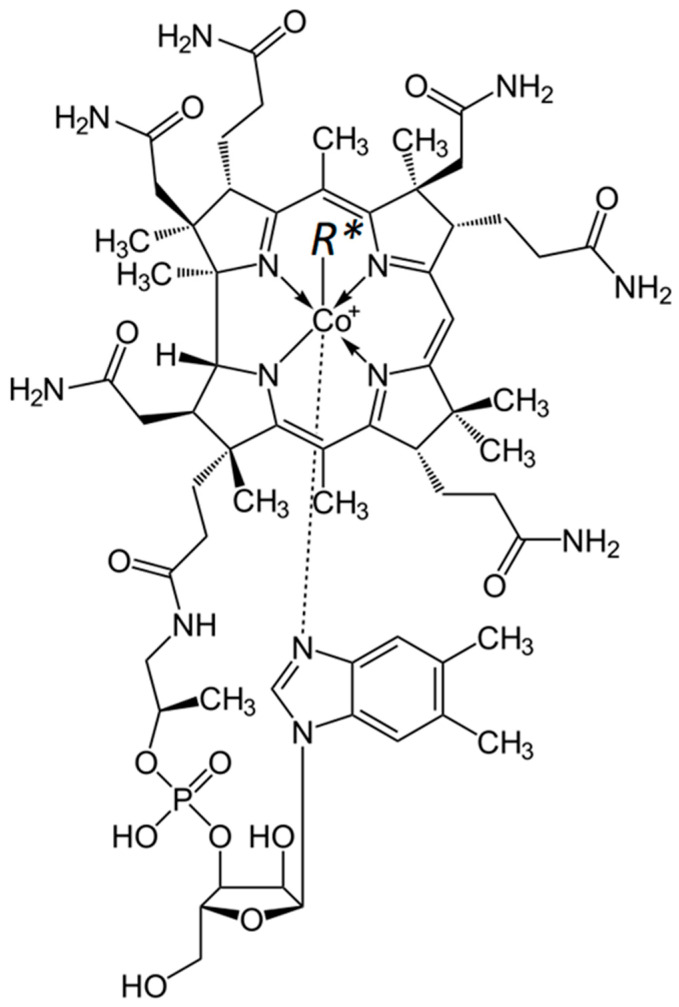
Various forms of vitamin B12; R* (depending on chemical form): methylcobalamin (MeCbl), adenosylcobalamin (AdCbl), hydroxocobalamin (OHCbl), and cyanocobalamin (CNCbl) (author’s own engraving based on [2,6,18]).

Vitamin B12 is a general name for a group of cobalamins (cobalt corrinoids) with a similar chemical structure and similar physiological functions. Vitamin B12 belongs to a group of compounds containing a corrin system, built from four reduced pyrrole rings and a centrally located cobalt atom. Vitamin B12 consists of four basic chemical forms of cobalamin: methylcobalamin (MeCbl), adenosylcobalamin (AdCbl), hydroxocobalamin (OHCbl), and cyanocobalamin (CNCbl). These forms differ in the cobalt substituents (Figure 1). MeCbl, AdCbl, and OHCbl are B12 vitamins of natural origin. They are bioidentical to the forms found in the human body. CNCbl is a synthetic vitamin B12 [14,17,18,19].

Each molecular variant of vitamin B12, derived from food or supplements, is initially reduced in the human body to cobalamin, which is converted into active intracellular forms of vitamin B12 (i.e., MeCbl and AdCbl) during subsequent metabolic processes [17]. Hydroxocobalamin is a reserve form of vitamin B12, which is stored in various spaces of the body, mainly in the liver. OHCbl, after being released from internal stores, is converted into active coenzymatic forms in target cells [18,19]. Vitamin B12 performs important metabolic functions in the body. It is a coenzyme, essential in methylation reactions, including the methylation of homocysteine to methionine and the conversion of methylmalonyl-CoA to succinyl-CoA. As a component of complex enzymatic systems, cobalamins participate in many key endogenous transformations, including, among others, the synthesis of nucleic acids, membrane phospholipids, neurotransmitters, and the proper functioning of the myelin sheaths of nerve fibers. As a factor involved in many processes, it regulates the functioning of various systems and organs of the body. Vitamin B12 is essential for hematopoietic processes and has a significant effect on the proper functioning of the nervous system (among other things, it is necessary for the construction of myelin sheaths of nerve fibers and participates in the creation of neurotransmitters). This vitamin regulates the metabolic transformations of fats and carbohydrates, participates in the synthesis of proteins and the metabolism of purines and pyrimidines, and is essential in the processes of the transformation of folic acid into biologically active forms [4,14,17,20,21,22,23,24].

### 2.1. Vitamin B12 in Historical Perspective

The discovery of vitamin B12, understanding and explaining the role of this vitamin in human metabolism, and the effects of its deficiency span over 100 years of research and observation. The first descriptions of red blood cell disorders, then called pernicious anemia, date back to the first half of the 19th century. It was already suspected then that this pathology was related to some kind of deficiency of essential nutrients [25]. In 1920, it was proven that feeding liver to exsanguinated dogs accelerates their recovery [26], and in 1926 experimental attempts were made to identify the component responsible for this effect [27]. In 1948, Rickes et al. [28] isolated a pure, crystalline compound of red color from the liver, which in doses of several micrograms prevented the occurrence of anemia. It was determined that this compound contained phosphorus and cobalt. Initially, it was called vitamin B12, and later cobamine [28,29]. The structure of vitamin B12 was described in 1956 by Dorothy Hodgkin (based on X-ray crystallography images). This description became the basis for the final determination of the molecular structure and chemical formula of vitamin B12 in 1964 [30,31]. At least two Nobel Prizes were awarded for achievements resulting from research on vitamin B12. In 1934, George Hoyt Whipple (University of Rochester), George Richards Minot (Harvard University), and William Parry Murphy (Harvard University) were awarded the Nobel Prize in Physiology/Medicine “for their discoveries concerning liver therapy in cases of anemia” (for proving that it is possible to cure a serious blood disease called pernicious anemia by means of liver extracts) [32]. In 1964, Dorothy Crowfoot Hodgkin (Cambridge University) received the Nobel Prize in Chemistry “for the elucidation of the structure of important biochemical compounds” [33].

### 2.2. Natural Food Sources and Vitamin B12 Absorption

Animals, including humans, are unable to synthesize cobalamin. Vitamin B12 is also not produced by plants (with a few exceptions). Only bacteria, including intestinal bacteria, and other single-cell organisms have the ability to produce it [4,34]. Until recently, it was commonly believed that the synthesis of vitamin B12 by the body’s own intestinal bacteria is of little importance from the point of view of its availability to this organism, because it takes place outside the main area of active intestinal absorption of this vitamin [4,34]. Kurpad et al. [35] estimated the bioavailability of vitamin B12 from the colon at 7% ± 5% (of the administered dose) over 4 h.

To cover the daily requirement for vitamin B12, it must be supplied from outside, preferably in the form of food from a balanced diet. The main dietary sources of vitamin B12 in the daily diet are animal products, such as meat products (mainly offal), milk and dairy products, eggs, fish, and crustaceans [36,37]. Plant foods contain virtually no vitamin B12. Small amounts can be found in the fruiting bodies of edible fungi and edible algae [38] and bacteria associated with plants [39]. It should be noted, however, that although such foods do not usually cover the daily requirement for cobalamin, products derived from plants such as purple algae (*Porphyrin* sp.), green algae (*Enteromorpha* sp.), and fermented soy products (e.g., douchi and tempeh) and cap mushrooms can be a source of this vitamin in a vegan diet [36].

In natural food sources, cobalamin occurs in the form of complexes with proteins. In the gastrointestinal tract, vitamin B12 protein complexes are degraded by pepsin and the acidic environment of the stomach [17,18,40]. The processes of absorption and transport of vitamin B12 in body fluids and between cells are very complex. They involve transport (escort) proteins, i.e., intrinsic factor (IF; also known as Castle’s factor), haptocorrin (HC), and transcobalamin (TC), their respective membrane receptors [41], and intracellular chaperones [42,43]. The absorption process of vitamin B12 begins in the oral cavity where, under the influence of saliva, it is released from food and bound in a complex with haptocorrin (HC). The vitamin B12/haptocorrin complex is broken down in the duodenum by proteolytic pancreatic enzymes. The released vitamin B12 is passed to the stomach where it is then bound in a complex with intrinsic factor (IF), a mucoprotein secreted by gastric parietal cells. The vitamin B12/IF complex enters the mucosal cells in the distal ileum by receptor-dependent endocytosis. This complex is then degraded in lysosomes and the released vitamin B12 is bound to a nonglycosylated carrier protein—transcobalamin (TC). In the form of a complex with TC, vitamin B12 is transported in the blood to target cells and the liver. In the target cells, vitamin B12 is processed into active forms (MeCbl and AdCbl). In the liver, it is stored in the form of hydroxocobalamin [22]. In blood, about 75% of cobalamin occurs in the form bound to transcobalamin I, and 25% occurs in the form bound to transcobalamin II (holotranscobalamin; holoTC). Only the holoTC form is bioavailable and can be used by cells [44,45]. Enterohepatic circulation and renal reabsorption of cobalamin with the participation of the receptor protein (megalin) contribute to the sparing management of this vitamin and cause the excretion of excess absorbed cobalamin to be significantly limited. Vitamin B12 is stored in tissues, mainly in the liver, kidneys, and muscles. Due to this phenomenon, clinical symptoms of cobalamin deficiency appear only after several years of insufficient supply of this vitamin [21,23,46,47].

### 2.3. Vitamin B12 Deficiency

Both the limited group of food products that are a source of vitamin B12 and the complicated mechanisms of its absorption may underlie cobalamin deficiency. Both of these problems mean that cobalamin deficiency is mainly caused by its insufficient intake (a common complication of a vegetarian or vegan diet) or intestinal absorption disorders of vitamin B12, which occur in the course of diseases of various origins (Table 1) [41]. Also, physiological processes related to the aging of the organism [44], pharmacotherapy with certain drugs (such as metformin, proton pump inhibitors of gastric parietal cells (including omeprazole, pantoprazole), and histamine H2 receptor antagonists, e.g., ranitidine), or resection of parts of the gastrointestinal tract (stomach, small intestine) [4,24] may result in absorption disorders of this vitamin of varying intensity. All these patients are considered to be at increased risk of developing vitamin B12 deficiency.

Due to the involvement of vitamin B12 in many enzymatic processes, its deficiency results in the impaired function of many different organs and systems. It can also cause mental disorders and cognitive functions. In the hematopoietic system, the production of erythroblasts is impaired, which leads to their premature destruction in the bone marrow. Ineffective erythropoiesis leads to the formation of large, spherical erythrocytes, the lifespan of which is shortened due to their abnormal structure and lower resistance to mechanical damage and other environmental conditions. This pathology is called megaloblastic anemia. Symptoms include general symptoms of anemia (weakness, increased fatigue, difficulty concentrating, dizziness, rapid heart rate, shortness of breath, paleness) and symptoms from the digestive system, such as weight loss and loss of appetite, loss of taste, nausea, diarrhea, or constipation. Characteristic is a burning tongue, which becomes dark red, smooth, shiny, and enlarged. Vitamin B12 deficiency can also lead to changes in the gastrointestinal mucosa and damage to the nervous system, such as peripheral neuropathy, spinal cord degeneration, or optic neuropathy. This is associated with a disorder of purine base synthesis, which impairs the metabolism of nucleic acids, and disorders of myelin synthesis, which result in damage and subsequent atrophy of nerve fibers. For this reason, in vitamin B12 deficiency anemia, neurological symptoms such as tingling or pricking in the fingers, numbness in the hands and feet, or vibration sensation disorders may also occur. Vitamin B12 deficiency lasting longer than 3 months can lead to permanent damage to the nervous system and cognitive disorders and dysfunctions in the area of mental health (e.g., depression, memory disorders, dementia, psychosis). It should be noted that neurological symptoms resulting from vitamin B12 deficiency may develop for many years, while megaloblastic anemia may be asymptomatic for a long time and does not have to precede the occurrence of nervous system dysfunctions. The effect of vitamin B12 deficiency is hyperhomocysteinemia, which predisposes to the development and deepening of atherosclerotic changes and increases the risk of circulatory system diseases, nervous system diseases, and cancers [4,24,35,48,49].

### 2.4. Vitamin B12 Deficiency Diagnostics

Since there is no “gold standard” laboratory test for assessing vitamin B12 status in the body, the diagnosis of vitamin B12 deficiency is difficult, and the clinical picture is the most important element of this process [5]. Serum vitamin B12 concentration below the lower limit of the reference range (the reference range is related to the analytical method) is a strong indicator of deficiency when it correlates with the symptoms observed in the patient. However, it should be noted that symptoms of cobalamin deficiency may also occur in people with normal vitamin B12 levels. This means that normal serum vitamin B12 levels do not exclude its deficiency [5,24,50].

An additional difficulty in diagnosing cobalamin deficiency occurs in patients taking oral vitamin B12 supplementation who do not have absorption disorders. In these patients, serum cobalamin concentration may be within the reference range or even exceed B12, despite the occurrence of characteristic, mainly neurological, symptoms of vitamin B12 deficiency [5].

In difficult diagnostic cases of suspected vitamin B12 deficiency, when laboratory cobalamin test results do not correlate with clinical symptoms, it may be helpful to determine serum methylmalonic acid (MMA) or homocysteine levels [51,52,53]. However, this strategy also has certain limitations. The concentration of homocysteine in serum may be increased also with deficiency of folic acid, vitamin B6, vitamin B2, impaired renal function, hypothyroidism, and during therapy with various drugs [54,55]. Also, the concentration of serum MMA may be modified with various drugs, regardless of vitamin B12 status. and is dependent on proper kidney function [56,57,58,59].

Another problem in diagnosing vitamin B12 deficiency and monitoring the effectiveness of therapy is that cobalamin levels measured by different laboratory methods in the same blood sample may differ. This is due to the fact that medical laboratories use different formats of immunochemical methods certified for in vitro diagnostics (IVD). Immunochemical methods differ in sensitivity and specificity, which may be reflected in the final results of the measurements performed. For this reason, if monitoring is necessary, tests performed by the same method (ideally in the same laboratory) should be used [60,61,62].

### 2.5. Therapy of Vitamin B12 Deficiency

Therapeutic treatment of vitamin B12 deficiency is aimed at supplementing the status of this vitamin in the body, which should ultimately lead to the withdrawal of clinical symptoms caused by its deficiency. The form and duration of therapy are strictly dependent on the cause of the deficiency, the physiological status of the patient, their clinical condition, and the extent of cobalamin deficiency (Table 2) [5].

If vitamin B12 deficiency is caused by an insufficient supply of cobalamin in the diet (with its absorption from the gastrointestinal tract not impaired), it is necessary to modify the diet and introduce products rich in vitamin B12, oral supplementation with vitamin B12 preparations or possibly supplementation in the form of intramuscular injections of this vitamin. In some countries, vitamin B12 is also available in sublingual form. Each form of vitamin B12 administration has advantages and disadvantages (Table 3) [6,63,64,65,66,67,68].

If vitamin B12 deficiency is caused by disease processes that impair its absorption, it is necessary to constantly and systematically supplement the cobalamin deficiency parenterally for the rest of the patient’s life. The frequency and amount of the dose administered to the patient depends on the type and intensity of the symptoms. The method of administration and its duration are determined by the cause of the deficiency and the established therapeutic target [5,6].

It is also assumed that cobalamin supplementation is necessary until the symptoms of deficiency disappear (in the case of dietary modification) or must be introduced permanently if the diet does not cover the daily requirement for vitamin B12, which is usually the case when following a vegan/vegetarian diet. Also, permanent impairment of the absorption of this vitamin in the digestive tract requires its constant supplementation in a way that allows the patient’s body to meet the needs for cobalamin. Maintenance (preventive) supplementation should be carried out in a way that causes the least discomfort to the patient while maintaining the effective availability of the administered dose [5,6,69].

Detailed guidelines for the regimen and intensity of dosing may vary across countries. Also, the availability of individual forms of vitamin B12 and the forms of therapeutic preparations used may not be the same [5,69,70,71,72,73].

### 2.6. Vitamin B12 Hypersensitivity

Although hypersensitivity to vitamin B12 is not common, it may be a significant clinical problem due to the fact that the need for therapy with this vitamin concerns a relatively large group of patients (e.g., in Great Britain and the United States of America it is estimated that vitamin B12 deficiency occurs in about 20% of people over 60 years of age) [8].

In order to estimate the scale of the phenomenon, El Rhermoul et al. [8] performed skin prick tests with cyanocobalamin (1 mg/mL) and hydroxocobalamin (1 mg/mL) and intradermal tests with these substances (in two concentrations: 0.1 and 0.01 mg/mL) in 29 people treated with vitamin B12. In patients with negative skin test results, a drug provocation test with vitamin B12 was performed. As a result of the tests, it was observed that 62% (18 of 29 patients) of the examined people developed an immediate reaction (including anaphylactic reactions: 1 after oral administration; 7 after intramuscular administration) and 13% a delayed reaction to both forms of cobalamin. It was noted that the majority of patients reacted to intramuscular administration of cobalamin. Only one patient did not tolerate the oral form. It seems, therefore, that the route of vitamin B12 administration is an important factor in determining the probability of developing a hypersensitivity reaction to cobalamin. Parenteral administration (e.g., intramuscular/subcutaneous/intravenous) increases the risk of this complication [8,9,74,75,76,77]. This observation seems to be confirmed by reported descriptions of cases of hypersensitivity to vitamin B12, which, mostly, document various types of skin reactions occurring at different times after intramuscular injection of this vitamin preparation [9,74,75,76,77]. In the context of cobalamin therapy, it seems important to note that hypersensitivity to vitamin B12 administered parenterally with simultaneous tolerance of oral preparations of this vitamin is also possible [18,19,20,21,22,23,24,25,26,27,28,29,30,31,32,33,34,35,36,37,38,39,40,41,42,43,44,45,46,47,48,49,50,51,52,53,54,55,56,57,58,59,60,61,62,63,64,65,66,67,68,69,70,71,72,73,74,75,76,77,78,79].

#### 2.6.1. Allergic Cross-Reactivity of Cobalamins

Since vitamin B12 can occur in various forms, the possibility of an allergic cross-reaction to different cobalamin variants seems likely, but based on the available literature data [80,81,82,83,84], this hypothesis cannot be unequivocally confirmed or ruled out. The available literature contains descriptions of cross-reactions to different cobalamin variants [80,81] as well as cases of hypersensitivity to one form of vitamin B12 with simultaneous tolerance to other chemical forms [82,83]. The cause of this phenomenon has not yet been explained.

#### 2.6.2. Duration of Supplementation and the Risk of Hypersensitivity to Vitamin B12

In many reported clinical cases of hypersensitivity reactions to vitamin B12 [9,10,11,12,13,82,83,84,85], it is noteworthy that allergic responses to the administered cobalamin developed after a long period of systematic therapy with cobalamin injections (different chemical forms). It seems likely, therefore, that long-term therapy is a significant risk factor for allergy to cobalamins used in the treatment of vitamin B12 deficiency. The question also arises whether a patient allergic to one form of cobalamin can develop hypersensitivity to another form of vitamin B12 during long-term therapy. As indicated by the observations of Heyworth-Smith and Hogan [85], it seems likely that allergy to one chemical form of vitamin B12 may predispose to allergy to other forms of this vitamin at a later time. These researchers [85] described the case of a 45-year-old female patient with intradermal hypersensitivity to hydroxocobalamin confirmed by intradermal tests, whose symptoms of cobalamin allergy increased with the duration of therapy. The patient was initially replaced with cyanocobalamin, which was initially well tolerated. However, during the 12-month therapy, the patient developed a rash at the site of the cyanocobalamin injection [85].

#### 2.6.3. Route of Administration and the Risk of Hypersensitivity to Vitamin B12

Vitamin B12 can be supplemented orally (if intestinal absorption is maintained) or by intramuscular or subcutaneous injection (when intestinal absorption is impaired). It should be considered whether the route of administration may be a risk factor for cobalamin allergy. In most cases of vitamin B12 hypersensitivity, various types of allergic reactions to parenterally administered cobalamins (intramuscular or subcutaneous) have been described [9,10,11,12,13,82,83,84,85]. Rhermoul et al. [8] emphasize that oral vitamin B12 supplementation does not seem to cause hypersensitivity reactions. Furthermore, it cannot be ruled out that hypersensitivity to tableted vitamin B12 may be caused by an allergy to the excipients used in the tablet formulation (e.g., polyethylene glycol; PEG) and not to the active substance (cobalamins) [8].

#### 2.6.4. Cobalt Allergy and a Risk Vitamin B12 Hypersensitivity

Due to the fact that vitamin B12 contains cobalt as the central atom of the molecule, the problem of hypersensitivity to cobalamin may also particularly concern people allergic to cobalt [86,87]. Pongcharoensuk and Thaiwat [88] reported a case of systemic, pigmentary, contact dermatitis (confirmed by histopathological examination) that occurred in a 55-year-old woman during a three-month oral supplementation of vitamin B12 (600 µg/day) due to neuropathic pain. Standard patch tests, performed according to the guidelines of the European Society of Contact Dermatitis (ESCD) [89] with the standard series and the cosmetic series, confirmed allergy to cobalt. Hyperpigmentation reactions occurred in the testing area at 48 and 72 h of testing. Since a relationship between cobalt allergy and vitamin B12 hypersensitivity cannot be ruled out, patients with known cobalt allergy are advised to remain vigilant when cobalamin supplementation is necessary [90,91].

#### 2.6.5. The Mechanism of Vitamin B12 Hypersensitivity

**Figure 2:**
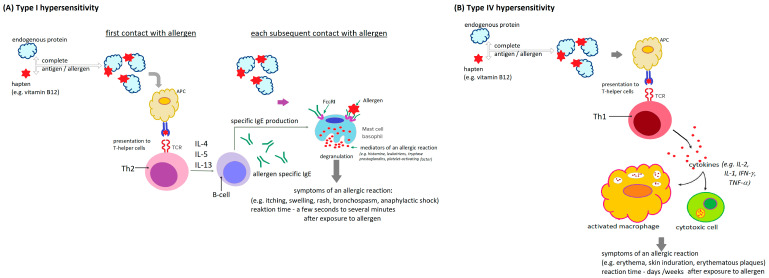
General scheme of type I (**A**) and type IV (**B**) hypersensitivity reactions to hapten (e.g., vitamin B12); APC—antigen-presenting cell, Th2—T helper cell type 2, TCR—T-cell receptor, IL—interleukin, Th1—T helper cell type 1, IFN-γ—interferon γ, TNF-α—tumor necrosis factor α, FcεRI—high-affinity type I receptor for immunoglobulin E [7,93,94].

Drug hypersensitivity reactions can be modeled in many different mechanisms. Both immunological and nonimmunological reactions are possible. Immunological drug hypersensitivity is mainly IgE-mediated reactions (immediate-type reactions) (Figure 2A) and late cellular reactions (Figure 2B). Nonimmunological drug hypersensitivity is mainly pharmacological interactions and pseudoallergies [7]. Drugs often do not have the characteristics of antigens but are haptens that, when combined with various endogenous carrier proteins, acquire the characteristics of complete antigens [92].

The mechanism of hypersensitivity to vitamin B12 has not been finally explained, and although some authors indicate an IgE-dependent basis for these reactions [78,82,95,96] (Type I (immediate) hypersensitivity reaction (Figure 2A)), histopathological examinations of samples from the skin lesions do not allow to unequivocally confirm this hypothesis or exclude the cellular nature (Type IV (late) hypersensitivity reaction (Figure 2B)) of the immune response to this vitamin. In both cases, vitamin B12 is probably a hapten, and intramuscular or subcutaneous administration favors a reaction to metabolically unchanged cobalamin administered in a short period of time in a large dose. This may explain the more frequent hypersensitivity to vitamin B12 after administration by injection. [88].

Interestingly, other, more atypical mechanisms are also discussed, which may involve skin bacteria from the *Propioni bacteriaceae* family [97,98]. According to the studies of Kang et al. [99], a high concentration of vitamin B12 in the pilosebaceous follicle promotes the production of porphyrins in Propionibacterium acnes colonies (current name *Cutibacterium acnes*). Porphyrins, undergoing the oxidation process on the skin surface, release proinflammatory substances, which ultimately promote the development of acne-like lesions [100,101,102,103,104,105], which may leave permanent scars [97]. This phenomenon seems to be promoted by supplementation with high doses of vitamin B12 or its long-term use [106].

Rhermoul et al. [8] also pointed out that the administration of vitamin B12 may be accompanied by various dermatological symptoms (itching and rash), diarrhea, fatigue, palpitations, or a feeling of swelling in the limbs, resulting from a rapid increase in the concentration of cobalamin in the blood. These symptoms can easily be confused with a hypersensitivity reaction. These authors suggest that vitamin B12 allergy should be diagnosed with caution because it is a rather rare problem [8], and overdiagnosis of cobalamin allergy may entail unnecessary therapeutic restrictions, which may have adverse effects on the patient.

### 2.7. Vitamin B12 Desensitization Protocols

Vitamin B12 therapy is usually long-term and there is no possibility of replacing it with an alternative form of treatment [107,108]. This makes the problem of hypersensitivity to vitamin B12 so important that, in addition to standard solutions, such as vitamin B12 injections with premedication [81], attempts are made to desensitize patients who require constant supplementation with this vitamin, and these actions seem to bring the expected results [9,10,11,12,13,14].

**Figure 3:**
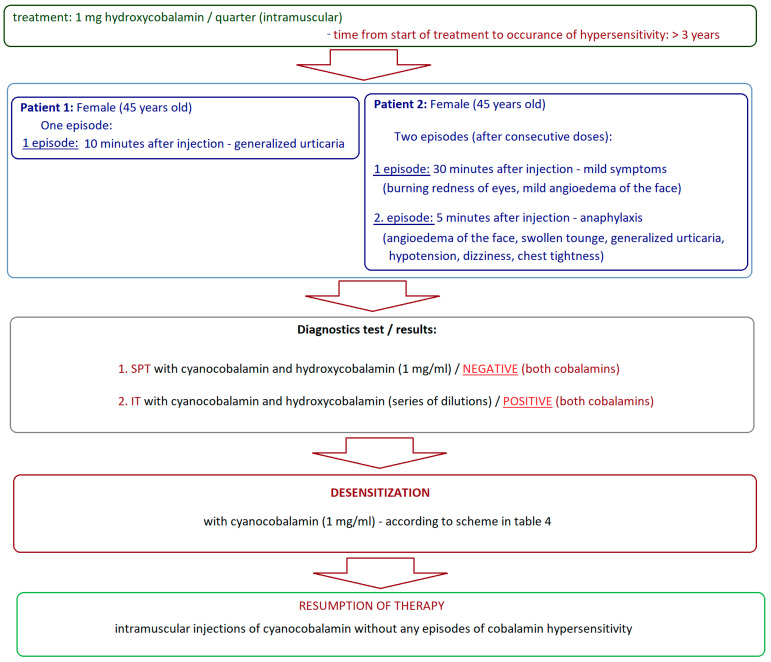
Clinical cases of diagnostic and therapeutic procedures in hypersensitivity to vitamin B12 with the desensitization procedure according to Caballero et al. [10]; SPT—skin prick tests; IT—intradermal tests.

According to published data, the first successful cyanocobalamin desensitization with a long-lasting effect was performed by Caballero et al. [10] in two patients suffering from pernicious anemia, who developed a hypersensitivity reaction after more than 3 years of therapy with quarterly intramuscular injections of vitamin B12 (Figure 3).

Both patients were desensitized in hospital conditions according to the same scheme (Table 4). After completion of desensitization, both patients continued intramuscular injections of cyanocobalamin, showing good tolerance.

Vitamin B12 desensitization, according to the same protocol (Table 4), was performed by Kartal et al. [9]. This team desensitized a 39-year-old vegetarian woman with vitamin B12 deficiency who, during approximately 10 months of intramuscular cyanocobalamin supplementation (1 injection; 10 mg per month), developed an itchy rash and widespread urticarial lesions over the entire body approximately 30 min after the 10th injection. The patient had no previous history of atopy. The woman underwent skin prick tests (SPT) with cyanocobalamin and hydroxocobalamin (both at a concentration of 1 mg/mL). The solutions used in the tests were free of dyes and preservatives. Positive results were obtained for both forms of vitamin B12. The reaction to cyanocobalamin was stronger. The patient’s desensitization according to Caballero et al. [10] (Table 4) was conducted without premedication and proceeded without any adverse effects. After completing the entire desensitization cycle, the woman underwent a skin prick test with cyanocobalamin (1 mg/mL), the result of which was negative, which, according to the authors, confirmed the efficacy of desensitization to vitamin B12 [9]. It seems surprising, however, that Kartal et al. [9] did not assess the efficacy of this desensitization in clinical conditions, after therapeutic administration of vitamin B12, or the long-term maintenance of the achieved results. This lack of important data significantly limits the conclusions as to the actual efficacy of this therapeutic intervention.

**Figure 4:**
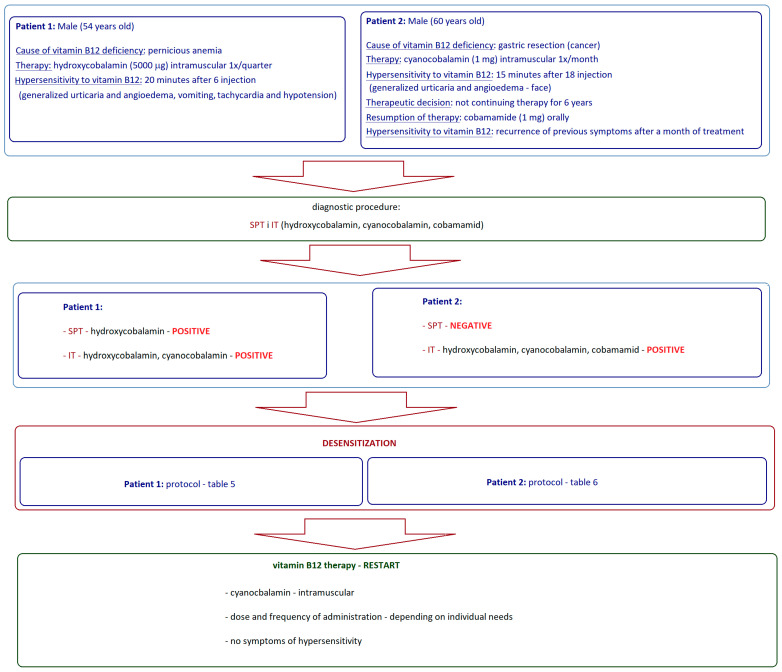
Clinical cases of diagnostic and therapeutic procedure in hypersensitivity to vitamin B12 with the desensitization procedure according to Costa et al. [11]; SPT—skin prick tests; IT—intradermal tests.

Two different schemes of vitamin B12 desensitization, individually tailored to the patient, were presented by Costa et al. [11]. Two patients (Figure 4) with vitamin B12 deficiency who developed hypersensitivity reactions during treatment with commercially available vitamin B12 were included in the intramuscular desensitization protocol [11].

Both patients underwent diagnostics and cyanocobalamin desensitization was implemented according to personalized protocols (Table 5 and Table 6), according to the test results and clinical symptoms observed in the patient). Desensitization was performed in the Day Unit, under medical supervision, with venous access maintained for up to 6 h after the last administration of each day. A commercial preparation of intramuscular cyanocobalamin (1000 µg/mL) was used, from which the required dilutions were prepared. No adverse effects were observed in any of the patients during this immunotherapy [11].

After desensitization, both patients were reintroduced to intramuscular cyanocobalamin according to individual indications (i.e., Patient 1: 500 µg 15/15 days for approximately 8 years; Patient 2: 1 mg monthly for 4 years). In both patients, normal vitamin B12 levels were achieved, and symptoms of its deficiency disappeared. Cobalamin therapy proceeded without adverse reactions that could result from hypersensitivity to vitamin B12 [11].

All the above protocols of vitamin B12 desensitization [9,10,11,12] are long procedures (duration from 3 to 49 days). Since discontinuing vitamin B12 supplementation for such a long time may have an adverse effect on the clinical condition of the patient, shorter, effective schemes of desensitization with this vitamin are sought. Alves-Correia et al. [13] proposed a short, 2.5 h long, cyanocobalamin desensitization scheme (Table 7), which they developed based on the solutions presented earlier [9,10,11,12,13].

Desensitization according to the protocol in Table 6 was performed in a hospital setting in a 61-year-old man with vitamin B12 deficiency who had been treated for 5 years with intramuscular injections of vitamin B12 preparations (cyanocobalamin at a dose of 1 mg/mL or cobamamide at a dose of 10 mg/2ml; 1 dose every 2 months) without any adverse effects until the last administration [13]. Two hours after the last administration of cyanocobalamin, the man developed angioedema of the face and hands with generalized itching and urticaria. The patient underwent SPT with cyanocobalamin and cobamamide (1 mg/mL and 5 mg/mL, respectively), the results of which were negative. Intradermal tests were performed with cyanocobalamin and cobamamide. Dilutions of 1:1000, 1:100, 1:10, and 1:1 were used for both tested substances. A positive result was observed in the immediate reading after 20 min, at a concentration of 1:10 for both cyanocobalamin (wheal—10 mm, erythema and pruritus) and cobamamide (wheal—8 mm, erythema, pruritus, periorbital angioedema). There were no late reactions. Intradermal solvent tests were negative. The patient also had asthma and allergic rhinitis. The total IgE concentration was 1440 kU/l, and the results of skin prick tests with house dust mites, olive pollen, and cat dander were positive [13]. During desensitization (according to the scheme as in Table 7), the patient was given a total of 9 subcutaneous injections, with a total cumulative dose of 1010 μg of cyanocobalamin. Alves-Correia et al. [13] opted for subcutaneous injection of vitamin B12 as it is less painful for the patient than intramuscular injections of this vitamin [6]. No local or systemic adverse events were observed during desensitization. The patient was discharged from the hospital 4 h after the last injection. The medical care team contacted the patient within 24 h. Complete blood count and serum vitamin B12 concentration were normal. After desensitization, the patient resumed therapeutic vitamin B12 injections (1 mg every 2 months) with good therapeutic effect and without any adverse reactions. The results of intradermal cyanocobalamin tests, repeated 6 months after desensitization, were negative [13].

The next two short cyanocobalamin desensitization protocols, the standard—seven-hour (Table 8)—and the rush—two-hour (Table 9)—were presented by Meerbeke et al. [15,16]. As reported by the authors [15,16], the efficacy of both of these protocols was verified by them in their clinical practice. Meerbeke et al. [15,16] pay special attention to the ultra-short, two-hour desensitization cycle with subcutaneous injections of cyanocobalamin in increasing concentrations (Table 8) because, as they showed, it can be performed in an outpatient setting. The efficacy and safety of this protocol were documented by conducting effective, uncomplicated immunotherapy in a 35-year-old woman with confirmed hypersensitivity to vitamin B12, treated with cobalamin injections in the course of Lesniowski–Crohn’s disease [15,16]. The possibility of carrying out vitamin B12 desensitization as a one-day therapy in an outpatient setting seems to be an attractive solution both for the patient and for reducing the costs of this therapy [15,16].

### 2.8. Vitamin B12 Desensitization—Mechanism and Effectiveness

**Figure 5:**
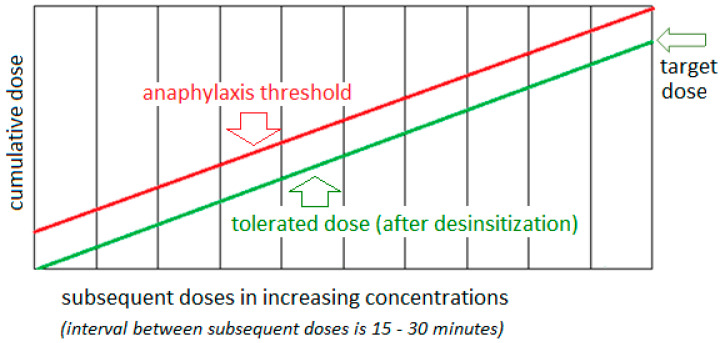
General assumptions of the protocols, goals, and effects of drug desensitization [109,111,112].

Drug desensitization is mainly used in patients who have experienced hypersensitivity reactions to a specific drug in the absence of alternative treatment options [109,110]. The general goal of drug desensitization is to induce tolerance to the drug, allowing a safe continuation of drug therapy. During desensitization, the patient should not experience side effects or, if such symptoms occur, they should be mild. The desensitization process is carried out according to a protocol that assumes a gradual increase in the drug dose until a therapeutic dose is reached, which is simultaneously associated with a gradual increase in the threshold concentration (which would cause anaphylaxis). It is known that mast cells and/or basophils can always release a certain amount of mediators during the desensitization procedure in response to the administered drug dose. It is therefore assumed that each subsequent dose administered induces stronger inhibition of effector cells and increases the threshold at which clinical symptoms are induced [111]. The initial dose (starting desensitization) is usually 10 to 10,000 times lower than the target dose. Subsequently, gradually increasing doses are administered to the patient at intervals of 15–30 min. The drug can be administered orally, sublingually, by intramuscular injection, subcutaneous injection, or by intravenous infusion with a gradually increasing flow, until the intended target dose is achieved (Figure 5) [112].

Desensitization is a recommended therapeutic strategy, especially when drug hypersensitivity occurs via an immediate IgE-dependent hypersensitivity reaction (type I hypersensitivity reaction). However, this therapy cannot be ruled out for non-IgE-dependent reactions, late-type IV cellular reactions, as well as nonimmunological reactions [112]. The drugs that most frequently require desensitization include antibiotics, anticancer drugs, antituberculosis drugs, and nonsteroidal anti-inflammatory drugs [109,111,112].

Depending on the drug, phenotype, and endotype of hypersensitivity reactions occurring in the patient and other individual characteristics, different desensitization protocols are used. Protocols usually differ in the starting dose, target dose, number of doses required, time intervals between doses, escalation of drug concentration from dose to dose, and route of administration. Rapid protocols, slow protocols, single- and multi-component protocols, and others are known [109,110,111,112]. In the case of sensitizing drugs, there are usually no standard desensitization protocols. Such a situation requires an individual approach to the patient each time. Such a strategy was also adopted by the authors of the previously cited various vitamin B12 desensitization protocols [9,10,11,12,13,14,15,16].

**Figure 6:**
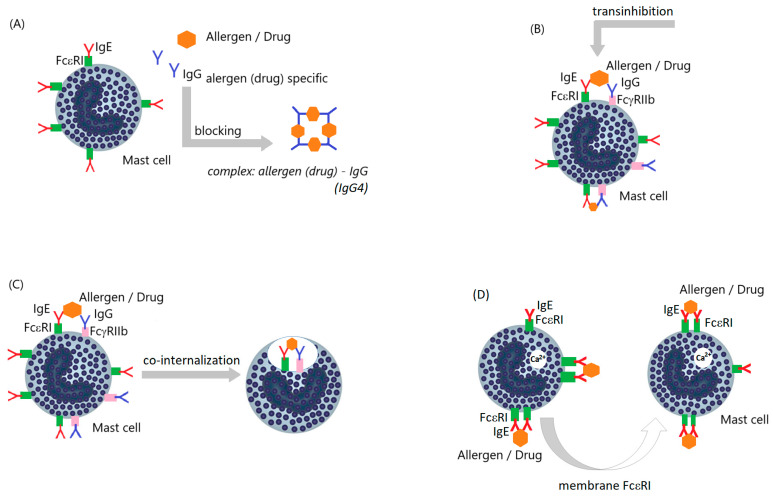
Probable mechanisms of desensitization to drugs (here vitamin B12): (**A**)—blocking of allergen/drug by specific IgG (mainly IgG4); (**B**)—blocking of FcεRI receptor bridging (simultaneous binding of allergen/drug by specific IgG on the mast cell surface); (**C**)—joint internalization of FcεRI and FcγRIIb receptors bridged by allergen/drug; (**D**)—rearrangement of FcεRI receptors as a result of administration of allergen/drug in increasing dose (blocking of internalization of bridged FcεRI receptors blocks mast cell degranulation) (author’s own figure based on [113,114]).

Currently, the mechanism of drug desensitization is not fully understood. It seems that many independent pathways blocking the immune response or nonimmunological pathways leading to the development of hypersensitivity reactions are involved in this process. Attention is paid primarily to the attenuation of various intracellular signals in target cells, e.g., rearrangement and disruption of the internalization of the allergen-bridged high-affinity IgE receptor (FcεRI), transinhibition or internalization of the FcεRI receptor with engagement of the low-affinity inhibitory receptor for the Fc region of immunoglobulin gamma (FcγRIIb), synthesis of blocking drug-specific immunoglobulins G4 (IgG4), alteration of signaling pathways in mast cells and/or basophils, and reduced calcium ion (Ca2+) influx into cells (Figure 6) [109,112,113,114].

The molecular mechanisms of vitamin B12 desensitization are not explained and none of the teams performing cobalamin desensitization [9,10,11,12,13,14,15,16] have undertaken to analyze these phenomena. In none of the previously described cases [9,10,11,12,13,14,15,16] was the efficacy of vitamin B12 immunotherapy assessed using independent methods (e.g., by assessing the concentration of specific IgG4 for cobalamins in the blood of desensitized patients after completing the therapy). In the opinion of these authors, the possibility of resuming and continuing cobalamin therapy by desensitized patients confirmed that desensitization was effective and the intended therapeutic effect was achieved [9,10,11,12,13,14,15,16].

## 3. Summary and Conclusions

Cobalamin is a coenzyme necessary for the proper activity of important metabolic pathways in animal organisms, including humans. Vitamin B12 deficiency results in impaired function of many organs and systems of the body, including the central nervous system. Each case of cobalamin deficiency absolutely requires supplementation. Since the mechanism of vitamin B12 availability in the diet is limited to animal products, and the mechanisms of its absorption are very complex, many different pathogenetic factors may underlie the deficiency. Supplementation with various cobalamin preparations is carried out orally or in the form of intramuscular or subcutaneous injections. The therapeutic strategy depends on the cause of the deficiency and is adapted to the individual requirements of the patient and the procedures available in a specific country [5,69,70,71,72,73,108]. Vitamin B12 supplementation is carried out until the deficiency is remedied, and if the factor causing the deficiency is not removed, continuous cobalamin supplementation is necessary. Oral therapy is a more convenient form for the patient, however, the deficiency is caused by absorption disorders, so it is necessary to administer cobalamin by repeated systematic intramuscular or subcutaneous injections of this vitamin [5,69,70,71,72,73,108]. Vitamin B12 therapy is considered to be rather safe, and hypersensitivity reactions have been described very rarely, usually after parenteral administration. However, because the need for chronic cobalamin supplementation may concern a significant number of people [8], and alternative therapy does not occur, this problem seems to be so important that several different strategies for desensitization of patients requiring cobalamin treatment, who have been diagnosed with hypersensitivity to vitamin B12 [9,10,11,12,13,14,15,16], which are presented above.

The analysis of the protocols of cobalamin desensitization and implementation, and the evaluation of the efficacy of this immunotherapy, which has been proposed and tested in clinical conditions by various authors [9,10,11,12,13,14,15,16], leads to the conclusion that cyanocobalamin desensitization is effective and probably leads to long-term desensitization to various cobalamins. This therapeutic strategy allows for the safe continuation of vitamin B12 therapy in patients who have previously experienced incidents of hypersensitivity to various forms of cobalamin, regardless of the route of administration and chemical form.

## Figures and Tables

**Table 1:** Selected diseases leading to the development of hypocobalaminemia and their pathogenetic mechanisms [4,22,24,41,44].

Disease	Mechanism of Impaired Absorption of Vitamin B12
Atrophic gastritis	Inflammation of the gastric mucosa leads to the atrophy of parietal cells and thus to the limitation of the secretion of hydrochloric acid and intrinsic factor. The disease may be associated with Helicobacter pylori infection or have an autoimmune basis.
Chronic pancreatitis	The insufficiency of the secretory function of the pancreas causes a deficiency of the digestive enzymes produced by it, which are necessary for the degradation of the cobalamin–haptocorrin complex in the duodenum.
Addison–Biermer disease	Autoimmune disease characterized by the presence of antibodies directed against intrinsic factor and/or against the gastric parietal cells that secrete it.
Lesniowski-Crohn’s disease	In the case of inflammation of the final section of the small intestine, the absorption of the cobalamin–intrinsic factor complex is impaired.
Imerslund–Gräsbeck syndrome	A genetic disease associated with a mutation in the genes encoding subunits of the Cubam receptor (cubilin or amnionless protein), which is essential for the absorption of the cobalamin–intrinsic factor complex into enterocytes.

**Table 2:** Main indications and routes of administration in vitamin B12 treatment [5,6].

Cause of Deficiency	Therapeutic Purpose	Recommended Administration Route
A diet deficient in vitamin B12 (based on plant ingredients) or excessive alcohol consumption	Prevention of vitamin B12 deficiency	Oral supplementation
	Replenishing vitamin B12 deficiency	Stage 1: intramuscular vitamin B12 therapy (to correct deficiency) Stage 2: oral supplementation (after symptoms of deficiency have subsided)
Related to various drug therapy (e.g., metformin, proton pump inhibitors, H2 histamine receptor inhibitors)	Prevention of vitamin B12 deficiency	Oral supplementation with vitamin B12 (as an adjunct to treatment that may result in cobalamin deficiency)
Impaired absorption of vitamin B12 in various diseases	Vitamin B12 deficiency replenishment and relapse prevention	Intramuscular injections administered for the rest of the patient’s life (in the case of diseases that cannot be cured) or until the cause disappears (in reversible cases, such as Helicobacter pylori infection or gastrointestinal parasites)
Chemical poisoning	Nitrous oxide toxicity	Intramuscular injections
	Cyanide poisoning	Intravenous administration of hydroxocobalamin

**Table 3:** Advantages and disadvantages of different routes of vitamin B12 administration [6,63,64,65,66,67,68].

Route of Administration of Vitamin B12	Advantages	Disadvantages
Oral	Self-administration No skills required Easy and most convenient Less painful than injections	The patient must be fasting The patient must be conscious The patient cannot have swallowing disorders, vomiting or diarrhea, or other diseases causing absorption disorders from the gastrointestinal tract
Sublingual	Ease of administration Fewer side effects (compared with injection) High bioavailability Rapid systemic impact Less painful than injections	The patient must be fasting The patient must be conscious The patient cannot have swallowing disorders, vomiting or diarrhea, or other diseases causing absorption disorders from the gastrointestinal tract
Intramuscular/subcutaneous	Absorption immediately after administration Uniform absorption Effective in emergency Absorption of the drug in an unchanged form Effective in patients with vitamin B12 absorption disorders Effective in cases where the patient has vomiting and/or diarrhea, or swallowing disorders Possible to perform in unconscious patients The patient does not need to be fasting while the drug is administered	Painful (intramuscular more than subcutaneous) Muscle mass may affect the effectiveness of treatment Nerve damage and paresis are possible The injection may only be performed by qualified personnel Greater risk of hypersensitivity reactions than after oral or sublingual administration

**Table 4:** Desensitization protocol with cyanocobalamin (1 mg/mL) according to Caballero et al. [10].

	Dilution *	Injection Volume (mL) **
Day 1	1:10,000	0.1; 0.3; 0.6
	1:1000	0.1; 0.3; 0.6
	1:100	0.1; 0.3; 0.6
	1:10	0.1; 0.3; 0.6
	1:0	0.1
Day 7	1:0	0.1; 0.3; 0.6
Day 21	1:0	0.1
Day 49	1:0	0.1

* Volume ratio—cyanocobalamin: diluent; ** subsequent doses on each day of desensitization are administered by intradermal injection at 15 min intervals.

**Table 5:** Cyanocobalamin desensitization protocol (Patient 1), according to Costa et al. [11].

Day	Solutions/Concentrations	Injection Volume (mL)	Cumulative Dose per Day
1	1:100 (10 µg/mL)	0.1; 0.2; 0.5	8 µg
2	1:10 (100 µg/mL)	0.1; 0.2; 0.4; 0.8	150 µg
3	1:1 (1000 µg/mL)	0.15; 0.25; 0.5	900 µg

The entire desensitization cycle lasts 3 days and includes 10 intramuscular injections. Subsequent injections were administered at 30 min intervals.

**Table 6:** Cyanocobalamin desensitization protocol (Patient 2), according to Costa et al. [11].

Day	Solutions/Concentrations	Injection Volume (mL)	Cumulative Dose per Day
1	1:500 (2 µg/mL)	0.5; 1.0; 2.0	7 µg
2	1:50 (20 µg/mL)	0.5; 1.0	30 µg
3	1:10 (100 µg/mL)	0.4; 1.0	140 µg
4	1:5 (200 µg/mL)	1.0; 1.5	500 µg
5	1:1 (1000 µg/mL)	0.5; 0.5	1000 µg

The entire desensitization cycle lasts 5 days and includes 11 intramuscular injections. Subsequent injections were administered at 30 min intervals.

**Table 7:** Protocol of rush cyanocobalamin desensitization according to Alves-Correia et al. [13].

Solutions/Concentrations	Injection Volume (mL)	Cumulative Dose per Injection Series
1:100/10 µg/mL	0.1 mL *; 0.3 mL *; 0.6 mL *	10 µg
1:10/100 µg/mL	0.1 mL *; 0.3 mL *; 0.6 mL*	100 µg
1:1/1000 µg/mL	0.1 mL **; 0.3 mL **; 0.5 mL	900 µg

* 15 min intervals between subcutaneous injections; ** 30 min intervals between subcutaneous injections.

**Table 8:** Standard (7 h) cyanocobalamin desensitization protocol [15,16].

Dose Number	Time Point/Hour of Procedure	Dilution	Concentration	Dose Volume (Subcutaneous Injection)	Administered Dose	Cumulative Dose
1	0 h	1:100	10 µg/mL	0.1 ml	1 µg	1 µg
2	0.5 h	1:100	10 µg/mL	0.3 ml	3 µg	4 µg
3	1 h	1:100	10 µg/mL	0.6 ml	6 µg	10 µg
4	2 h	1:10	100 µg/mL	0.1 ml	10 µg	20 µg
5	3 h	1:10	100 µg/mL	0.3 ml	30 µg	50 µg
6	4 h	1:10	100 µg/mL	0.6 ml	60 µg	110 µg
7	5 h	1:1	1000 µg/mL	0.1 ml	100 µg	210 µg
8	6 h	1:1	1000 µg/mL	0.3 ml	300 µg	510 µg
9	7 h	1:1	1000 µg/mL	0.6 ml	600 µg	1110 µg

**Table 9:** Rush (2 h) cyanocobalamin desensitization protocol [15,16].

Dose Number	Time Point/Hour of Procedure	Dilution	Concentration	Dose Volume (Subcutaneous Injection)	Administered Dose	Cumulative Dose
1	0 h	1:100	10 µg/mL	1.0 mL	10 µg	10 µg
2	0.5 h	1:10	100 µg/mL	1.0 mL	100 µg	110 µg
3	1 h	1:1	1000 µg/mL	0.1 mL	100 µg	210 µg
4	1.5 h	1:1	1000 µg/mL	0.3 mL	300 µg	510 µg
5	2 h	1:1	1000 µg/mL	0.6 mL	600 µg	1110 µg

## Abbreviations

The following abbreviations are used in this manuscript:

MeCbl	methylcobalamin
AdCbl	adenosylcobalamin
OHCbl	hydroxocobalamin
CNCbl	cyanocobalamin
